# Development and Formative Evaluation of a Narrative-Based Serious Game for Pregnancy Education: Mixed Methods Study

**DOI:** 10.2196/93571

**Published:** 2026-07-13

**Authors:** Yukihide Miyosawa, Ayako Furuya, Eri Okamura, Nanami Ogihara, Ayaka Onozato, Yu Miyazaki, Shunki Ando, Yasutaka Kimura

**Affiliations:** 1 Department of Pediatrics, Shinshu University School of Medicine, Asahi 3-1-1, Shinshu University School of Medicine Department of Pediatrics, Matsumoto, Nagano, 390-8621, Japan, 81 263372642; 2 Department of Medicine, School of Medicine, Shinshu University, Matsumoto, Nagano, Japan

**Keywords:** serious games, pregnancy education, maternal health, game-based learning, adolescent health, formative evaluation, mobile apps, narrative simulation, multiplatform deployment

## Abstract

**Background:**

Serious games are increasingly used in professional health education and maternal health promotion. However, most pregnancy-related digital interventions target specific behaviors and do not provide a comprehensive, longitudinal simulation of the pregnancy journey that incorporates psychosocial and administrative aspects.

**Objective:**

This study aimed to develop and evaluate a narrative-based serious game that simulates the chronological course of pregnancy and to assess its perceived educational usefulness, accessibility, and user acceptance across multiple platforms.

**Methods:**

We developed a 9-chapter interactive serious game covering pregnancy recognition, partner communication, public health consultation, mid-pregnancy and late-pregnancy checkups, and home preparation for childbirth. The game was collaboratively created by a pediatrician, 6 medical students, and a student illustrator using a low-cost visual novel engine (TyranoBuilder). It was released in April 2025 on iOS, Android, and Steam. A voluntary, anonymous postgame survey was conducted between April 2025 and January 2026. Descriptive statistics were used to summarize survey responses and platform analytics. This study was approved by the Ethics Committee of Shinshu University Hospital.

**Results:**

A total of 65 users completed the postgame questionnaire. Most respondents were aged 10 to 19 years (38/65, 58.5%) and female (55/65, 84.6%). Nearly half of the participants (30/65, 46.2%) completed the game within 1 hour. Gameplay evaluation scores (5-point Likert scale; 3=neutral or appropriate) were balanced: game length (mean 3.37, SD 0.96), difficulty (mean 2.84, SD 0.85), and interactivity (mean 3.31, SD 1.10). Educational outcomes were rated highly (5-point Likert scale; higher=more favorable): reduced anxiety (mean 3.84, SD 0.96), perceived educational usefulness (mean 3.98, SD 1.02), perceived knowledge acquisition (mean 4.06, SD 1.06), story empathy (mean 3.80, SD 1.11), and overall satisfaction (mean 4.05, SD 1.04). Across all platforms, the game achieved 925 cumulative downloads. iOS and Android downloads were predominantly from Japan, whereas Steam downloads were geographically diverse. Of the 21 Steam reviews, 20 (95.2%) were positive.

**Conclusions:**

A serious pregnancy education game developed through a low-cost clinician-student collaborative model demonstrated high perceived educational usefulness, balanced gameplay characteristics, and broad user acceptance, including substantial engagement among teenagers and international users. Narrative-based serious games represent an accessible and scalable approach to maternal health education. Further research using more rigorous evaluation designs is warranted to assess long-term educational and behavioral impacts.

## Introduction

Serious games are increasingly recognized as effective digital tools in professional health education. Systematic reviews and meta-analyses have demonstrated that serious games can improve knowledge, clinical skills, and learner engagement across a range of medical and nursing educational contexts, often outperforming traditional educational strategies [1,2]. User-centered development approaches, including the involvement of end users, such as students and clinicians, during the design process, have been shown to enhance the educational validity and acceptability of serious games [3].

The application of game-based or gamified educational interventions is expanding in maternal and perinatal health. A systematic review of gamification in prenatal care reported promising improvements in knowledge, attitudes, and preventive behaviors among pregnant women, although methodological heterogeneity remains a challenge [4]. Among adolescent pregnant women and young mothers, web-based digital tools have been associated with increased knowledge, self-efficacy, and improved maternal behaviors in a mixed methods evidence synthesis [5]. Serious mobile games targeting maternal and neonatal safety have demonstrated measurable benefits in community settings. For example, the Maternal and Neonatal Technologies in Rural Areas (MANTRA) game in rural Nepal significantly improved knowledge of maternal danger signs and geohazard preparedness in pre-post comparisons [6].

Serious games have also been used to address specific risky behaviors during pregnancy. A pilot randomized controlled trial of the Tobbstop game reported higher smoking cessation rates among pregnant smokers in the intervention group than among those receiving standard care [7]. Game-based antenatal breastfeeding education has been shown to improve maternal breastfeeding self-efficacy and early postpartum feeding outcomes [8]. Beyond targeting specific behaviors, digital interventions, such as mobile apps and SMS text message–based support, have demonstrated improved adherence to iron supplementation during pregnancy [9] and broader maternal health outcomes among pregnant women and mothers of young children [10].

Despite these developments, a recent scoping review of serious games for women’s health noted that most existing titles focus on short, narrowly defined tasks or single behaviors, with few games offering a longitudinal simulation of pregnancy or addressing the psychosocial and administrative aspects of prenatal care [11]. This gap highlights the need for interactive tools that enable users to experience the full trajectory of pregnancy, from early recognition to late-pregnancy checkups and birth preparation, within a narrative and emotionally engaging format.

Our previous study reported the successful development of a neonatal intensive care unit simulation game created collaboratively by clinicians and students using a low-cost, accessible development model. The game demonstrated high educational usefulness, emotional engagement, and broad acceptance among both health care and non–health care users [12]. These positive outcomes suggest that a similar student-assisted development approach may be applicable to pregnancy education.

To address the lack of comprehensive narrative-based pregnancy education tools, we developed a 9-chapter interactive serious game that simulates the chronological course of pregnancy. The game covers early pregnancy recognition, prenatal clinic visits, lifestyle guidance, communication with partners, administrative consultations, mid-pregnancy and late-pregnancy checkups, and home preparation for childbirth. Developed collaboratively by a pediatrician, 6 medical students, and a student illustrator using a low-cost tool (TyranoBuilder; STRIKEWORKS Inc), the game incorporates illustrated storytelling, interactive decision-making, Teaching Information and Prompt System (TIPS)–based microlearning, and bilingual (Japanese and English) support.

In this study, we aimed to evaluate the perceived educational usefulness, accessibility, and user acceptance of this pregnancy-themed serious game through postgame surveys, download analytics, and international user reviews. By building on prior findings in serious game–based health education, we sought to extend the field by providing a comprehensive experiential learning tool that spans the full course of pregnancy.

## Methods

### Study Design

This study adopted a formative mixed methods design, integrating the development of a serious pregnancy education game with a postrelease evaluation of users that included quantitative survey data and qualitative user feedback. The development period spanned from April 2024 to March 2025, and the game was released in April 2025 simultaneously on the iOS App Store, Google Play Store (Android), and Steam (Windows) platforms. The game was freely available without registration or cost, and users were recruited via an in-game prompt presented upon completing the game. A link to the anonymous postgame survey (hosted on Google Forms) was embedded within the game and displayed at the end of play. No systematic exclusion criteria were applied; all users who completed the game and consented to participate were eligible. The postgame survey was conducted between April 2025 and January 2026.

### Ethical Considerations

#### Compensation

No incentives or compensation were provided to participants.

#### Privacy and Confidentiality

Survey data were collected anonymously via Google Forms. No personally identifiable information was collected, and individual responses could not be linked to specific participants.

#### Informed Consent

Electronic informed consent was obtained from all participants at the beginning of the questionnaire prior to data collection.

#### Ethics Approval

This study was approved by the Ethics Committee of Shinshu University Hospital (approval number 6497).

### Development Team

The game was developed by a multidisciplinary team led by the first author, a pediatrician specializing in neonatology. Scenario development, including field interviews and contextual refinement, was supported by a pediatrician with experience in maternal health and clinical communication. Medical and administrative content were reviewed iteratively at multiple stages of development to ensure accuracy and consistency with current prenatal care practices.

The implementation team consisted of 6 medical students who were primarily responsible for programming, branching logic, and image processing, and 1 student illustrator who created all character illustrations, background art, and visual assets. No professional programmers were involved in the development process.

### Development Tools and Workflow

The game was created using TyranoBuilder, a visual novel engine that enables interactive storytelling, branching narratives, animation, and bilingual text support without requiring advanced programming expertise.

The game development workflow comprised seven sequential steps: (1) scenario design by the first author and a pediatrician with expertise in maternal health; (2) script implementation and branching logic by 6 medical students; (3) illustration production (all characters, backgrounds, and user interface elements) by the student team; (4) medical and administrative review by obstetricians, midwives, and public health nurses; (5) bilingual implementation (Japanese and English); (6) platform optimization for iOS, Android, and Steam; and (7) quality and usability testing by medical students and perinatal care professionals.

This workflow was designed to be reproducible in educational and academic settings with limited technical and financial resources. This resulted in a game that provides a fully illustrated, narrative-driven educational experience with an estimated playtime of approximately 1 hour and is freely accessible without advertisements or in-app purchases.

### Game Scenario

**Figure 1.**
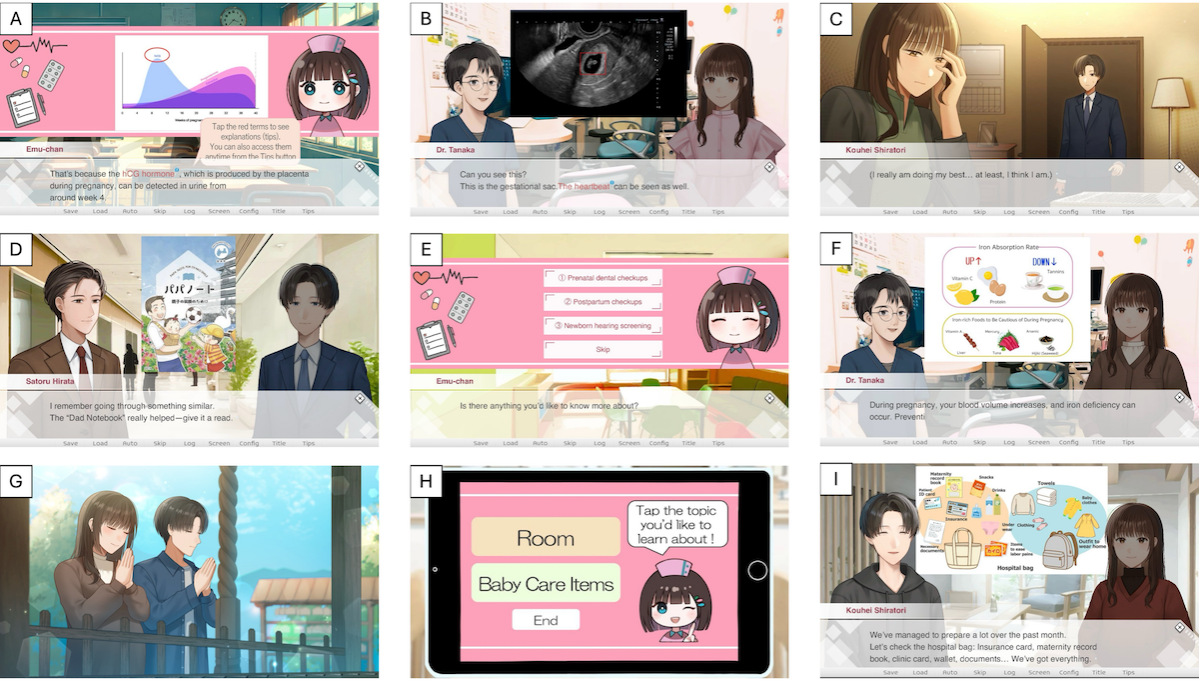
Overview of the pregnancy education serious game (chapters 1–9). Each panel shows a representative scene from one chapter of the game: (A) pregnancy recognition and confirmation (chapter 1), (B) the first obstetric and gynecological visit (chapter 2), (C) informal peer consultation (chapter 3), (D) partner communication and shared decision-making (chapter 4), (E) public health center consultations (chapter 5), (F) mid-pregnancy medical checkups (chapter 6), (G) daily life and safety during pregnancy (chapter 7), (H) preparation of the home environment (chapter 8), and (I) late-pregnancy checkups and delivery planning (chapter 9).

The game consists of a sequential 9-chapter storyline that simulates the course of pregnancy from early recognition to late-pregnancy checkups and preparation for childbirth. Each chapter includes illustrated scenes, interactive dialogue choices, and an optional TIPS that provides concise medical or administrative information (Figure 1).

The 9 chapters cover the following topics: pregnancy recognition and confirmation (chapter 1), the first obstetric and gynecological visit (chapter 2), informal peer consultation (chapter 3), partner communication and shared decision-making (chapter 4), public health center consultations (chapter 5), mid-pregnancy medical checkups (chapter 6), daily life and safety during pregnancy (chapter 7), preparation of the home environment (chapter 8), and late-pregnancy checkups and delivery planning (chapter 9).

### Postgame Survey

After completing the game, participants were invited to respond to an anonymous online questionnaire via Google Forms. The survey collected demographic information and evaluated gameplay experience and perceived educational usefulness. Survey items are summarized in Table 1.

**Table 1.** Postgame survey items.

Category and item	Response scale
Demographic variables	
Age group	Categorical
Sex	Categorical
Occupation	Categorical
Device used	Categorical
Playtime	<1 hour; 1-2 hours; 2-4 hours; >4 hours; Missing
Gameplay evaluation	
Game length	5-point Likert scale
Difficulty	5-point Likert scale
Gameplay interactivity	5-point Likert scale
Educational usefulness	
Reduced anxiety	5-point Likert scale
Educational usefulness	5-point Likert scale
Knowledge acquisition	5-point Likert scale
Story empathy	5-point Likert scale
Overall satisfaction	5-point Likert scale

For gameplay evaluation items (game length, difficulty, and gameplay interactivity), a score of 3 indicated an appropriate or balanced level (eg, appropriate length, appropriate difficulty, or a sufficient level of interactivity). Scores below 3 indicated the game was perceived as too short, too easy, or insufficiently interactive; scores above 3 indicated it was perceived as too long, too difficult, or overly interactive.

For educational usefulness items, higher scores indicated more positive evaluations, with 5 representing the most favorable response.

### Platforms and Analytics

The game was simultaneously released on the Apple App Store (iOS), Google Play Store (Android), and Steam (Windows).

Analytical data were extracted at a single predefined time point after release to ensure consistency across platforms. Platform-specific metrics were extracted from App Store Connect (Apple Inc), Google Play Console (Google LLC), and the Steamworks Developer Dashboard (Valve Corporation).

### Data Analysis

Survey data and platform analytics were summarized using descriptive statistics. For Likert-scale items, given the ordinal nature of the data, we report means, SDs, SEs, medians, and IQRs. Categorical variables are summarized as frequencies and percentages. Inferential statistics were not used. All analyses were performed using Microsoft Excel. Participants with missing responses on individual items were included in analyses for all items they completed and excluded only from item-specific calculations. No pre-post testing or between-group comparisons were performed, in accordance with the formative nature of the study.

Steam reviews were analyzed using inductive content analysis conducted by the first author (Y Miyosawa). All 21 reviews available on the Steam platform were included; reviews from iOS and Android platforms were not included, as these platforms do not provide a structured text-based review system equivalent to that of Steam. The analysis followed established procedures for qualitative content analysis [13,14], with initial open coding of all review texts, grouping of codes into subcategories, and abstraction into overarching thematic categories [13,14]. A second reviewer (AF) independently coded a subset of 5 reviews to verify the emerging categories, and any interpretive differences were resolved through consensus discussion to enhance trustworthiness. No formal coding software was used. In our sample, many reviews contained limited substantive content suitable for thematic analysis; only reviews offering substantive qualitative feedback were included in the thematic synthesis. The median length of Steam reviews has been reported as 19 words for positive reviews and 40 words for negative reviews [15].

## Results

### Participant Characteristics

A total of 65 users completed the postgame questionnaire; their characteristics are summarized in Table 2. The most common age group was 10 to 19 years (38/65, 58.5%), followed by 20 to 29 years (14/65, 21.5%), 40 to 49 years (7/65, 10.8%), 30 to 39 years (5/65, 7.7%), and ≥60 years (1/65, 1.5%). Most participants identified as female (55/65, 84.6%), with 4 (6.2%) identifying as male and 6 (9.2%) selecting a sex option not listed as female or male. The sex question offered 3 response options (female, male, and an open category); future surveys should include more inclusive response options in line with best practices for gender-diverse reporting. The largest occupational group was high school students (32/65, 49.2%), followed by non–health care professionals (14/65, 21.5%). Health care professionals and students collectively accounted for 11 (16.9%) participants, comprising physicians (3/65, 4.6%), nurses (3/65, 4.6%), medical students (2/65, 3.1%), nursing students (2/65, 3.1%), and other health care workers (1/65, 1.5%). Smartphones were the most commonly used device (42/65, 64.6%), followed by tablets (12/65, 18.5%) and PCs (7/65, 10.8%). Regarding playtime, 30 (46.2%) completed the game in less than 1 hour, 18 (27.7%) in 1 to 2 hours, 3 (4.6%) in 2 to 4 hours, and 4 (6.2%) in more than 4 hours. Ten participants (15.4%) did not report playtime; these participants were retained in all other analyses.

**Figure 2.**
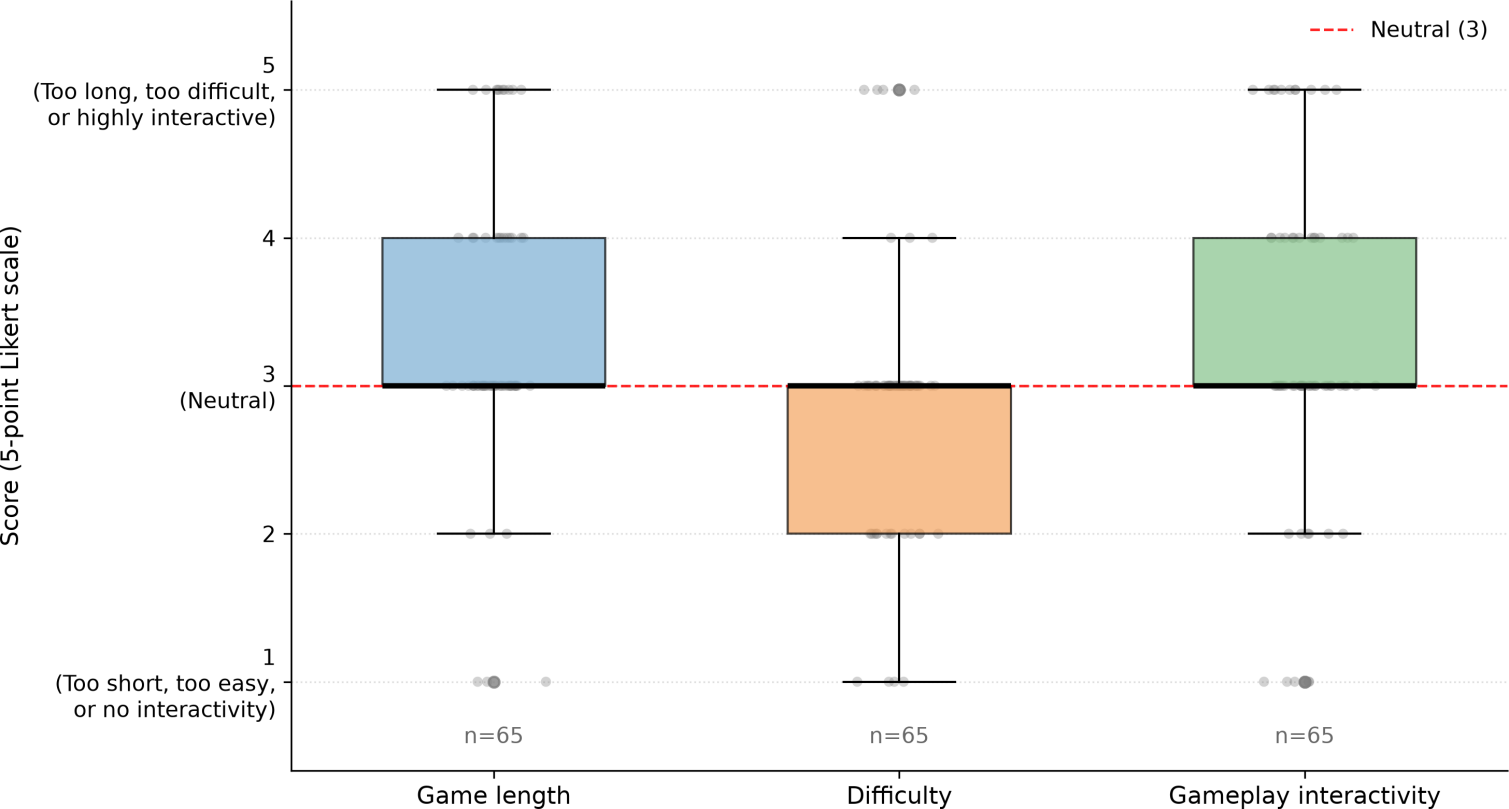
Gameplay evaluation scores (N=65) from the postgame survey. Scores are on a 5-point Likert scale (1-5), where 3 indicates neutral or appropriate. Whiskers extend to 1.5× IQR; gray dots indicate individual responses.

Figure 2 depicts gameplay evaluation scores from the postgame survey conducted among users (Japan and international users) of the pregnancy education serious game from April 2025 to January 2026.

**Table 2.** Participant characteristics (N=65).

Characteristics	Participants, n (%)
Age group (y)	
10‐19	38 (58.5)
20‐29	14 (21.5)
30‐39	5 (7.7)
40‐49	7 (10.8)
≥60	1 (1.5)
Sex	
Female	55 (84.6)
Male	4 (6.2)
Other	6 (9.2)
Occupation	
High school students	32 (49.2)
Other non–health care professionals	14 (21.5)
Physicians	3 (4.6)
Nurses	3 (4.6)
Medical students	2 (3.1)
Other health care workers	1 (1.5)
Nursing students	2 (3.1)
Other	8 (12.3)
Device used	
Smartphone	42 (64.6)
Tablet	12 (18.5)
PC	7 (10.8)
Other	4 (6.2)
Playtime	
<1 h	30 (46.2)
1‐2 h	18 (27.7)
2‐4 h	3 (4.6)
>4 h	4 (6.2)
Missing	10 (15.4)

Gameplay-related ratings were generally near the neutral midpoint of 3.0 on the 5-point scale. For game length, the mean score was 3.37 (SD 0.96) and the median score was 3 (IQR 3-4). For difficulty, the mean score was 2.85 (SD 0.85) and the median score was 3 (IQR 2-3). For gameplay interactivity, the mean score was 3.31 (SD 1.10) and the median score was 3 (IQR 3-4).

**Figure 3.**
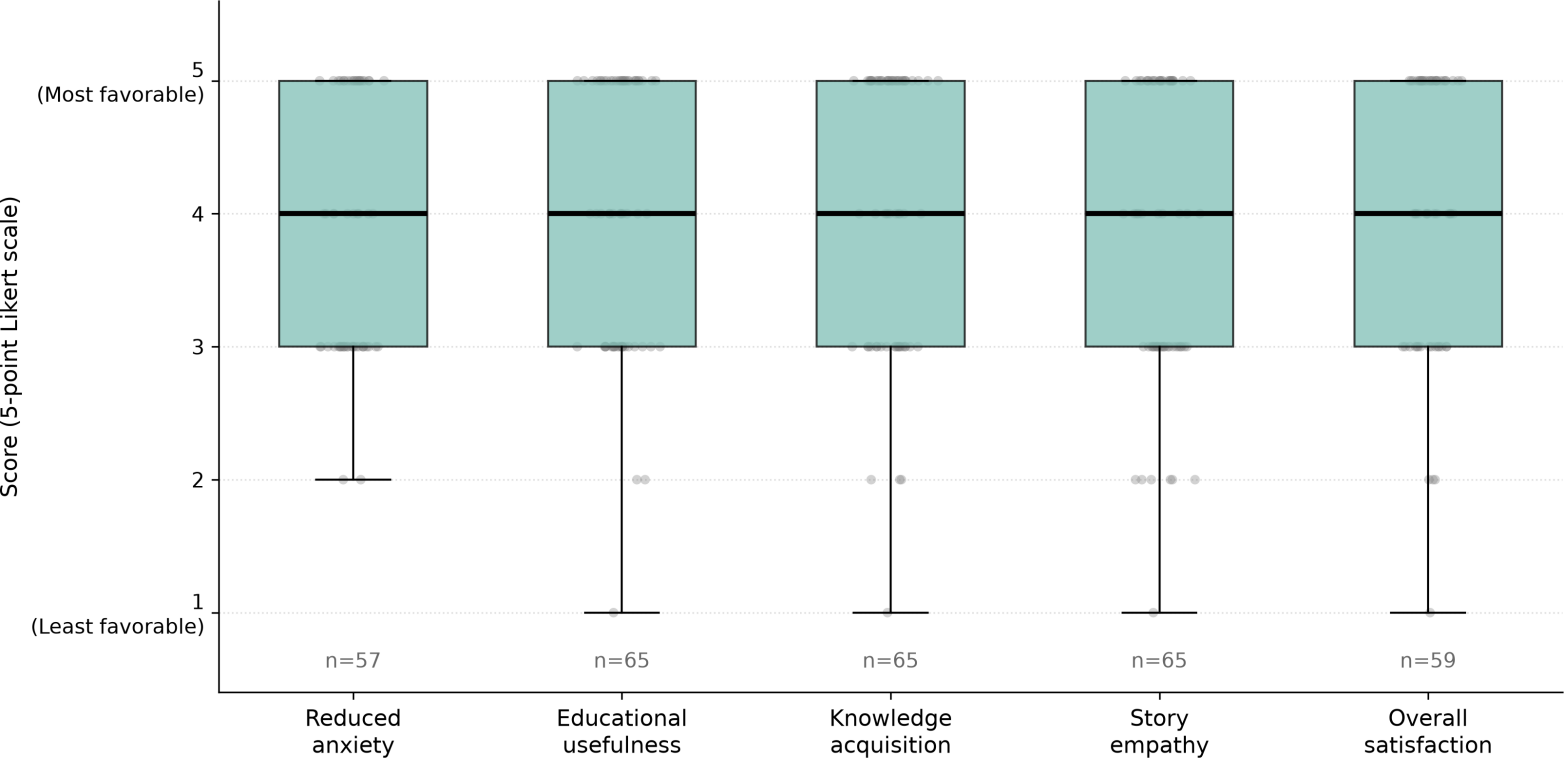
Perceived educational usefulness scores (n=57‐65) from the postgame survey. Scores are on a 5-point Likert scale (1-5), where higher scores indicate more favorable evaluations (5=most favorable). Whiskers extend to 1.5× IQR; gray dots indicate individual responses.

Figure 3 depicts perceived educational usefulness scores from the postgame survey conducted among users (Japan and international users) of the pregnancy education serious game from April 2025 to January 2026.

Perceived educational usefulness indicators received consistently favorable ratings. For reduced anxiety, the mean score was 3.84 (SD 0.96) and the median score was 4 (IQR 3-4; n=57). For perceived educational usefulness, the mean score was 3.98 (SD 1.02) and the median score was 4 (IQR 3-4; n=65). For perceived knowledge acquisition, the mean score was 4.06 (SD 1.06) and the median score was 4 (IQR 3-4; n=65). For story empathy, the mean score was 3.80 (SD 1.11) and the median score was 4 (IQR 3-4; n=65). For overall satisfaction, the mean score was 4.05 (SD 1.04) and the median score was 4 (IQR 3-4; n=59). Higher scores indicate more positive evaluations, with 5 representing the most favorable response. The reduced anxiety and overall satisfaction items had slightly lower response counts (n=57 and n=59, respectively) due to missing data.

### Subgroup Analysis by Age

An exploratory subgroup analysis by age (teenagers vs adults) did not reveal any substantial differences in gameplay evaluation or perceived educational usefulness between the 2 groups (Table S1 in Multimedia Appendix 1).

### Platform Downloads

Across iOS, Android, and Steam, the game had achieved 925 cumulative downloads by February 5, 2026. Statistics regarding downloads are summarized in Table 3 below.

On the iOS and Android platforms, downloads were predominantly recorded in Japan, whereas Steam downloads showed a more geographically diverse distribution.

**Table 3.** Country-level download distributions by platform.

Platform and country	Downloads, n (%)	
iOS (n=549)		
Japan	521 (94.9)	
United States	13 (2.4)	
China	4 (0.7)	
Canada	2 (0.4)	
Thailand	2 (0.4)	
France	2 (0.4)	
South Korea	2 (0.4)	
Other countries	3 (0.5)	
Android (n=102)		
Japan	80 (78.4)	
Other countries	22 (21.6)	
Steam (n=274)		
United States	96 (35)	
Japan	37 (13.5)	
Canada	16 (5.8)	
United Kingdom	14 (5.1)	
Germany	14 (5.1)	
Other countries	97 (35.4)	

### Steam Reviews

A total of 21 reviews were posted on Steam, of which 20 (95.2%) were positive. Content analysis of the 21 Steam reviews indicated that the majority were short and contained limited substantive content for thematic analysis, consistent with previously reported median review lengths on the Steam platform [15]. Among the subset of reviews containing substantive content (5/21, 23.8%), recurring positive themes included the game’s educational value and the accessibility of pregnancy-related information. Representative positive comments included: “Very educational and cute!”; “Learned a lot!!! its very good and provides thorough explanations on the experience of pregnancy”; and “Interesting and fun.” The single negative review cited technical usability concerns, including auto-advancing text messages, audio interruptions, and unexpected language switching, which were identified as priority targets for future improvement. iOS reviews comprised 2 five-star ratings and 1 one-star rating; Android received no reviews.

## Discussion

### Principal Findings

This formative study demonstrated that a serious pregnancy education game developed through a low-cost clinician-student collaborative model achieved broad user acceptance and high perceived educational usefulness. The game attracted a diverse user population, with particularly high engagement among teenagers, who constituted most survey respondents. Gameplay characteristics were rated near the neutral midpoint, reflecting balanced but modest interactivity, while indicators of perceived educational usefulness and overall satisfaction received consistently favorable ratings. The game achieved substantial international reach, with cumulative downloads from more than 40 countries across 3 platforms, and received predominantly positive ratings on Steam. Together, these findings suggest that narrative-based serious games may serve as accessible and scalable tools for maternal health education, even when developed with limited technical and financial resources.

A distinctive feature of our study was the high proportion of teenage users (58% aged 10‐19 years), most of whom were nonpregnant high school students. This demographic diverges from the game’s primary design intent of simulating the pregnancy experience for expectant individuals but underscores a valuable secondary use case: reproductive health literacy education among adolescents. Early exposure to accurate pregnancy-related information during adolescence may contribute to future health literacy and informed decision-making, consistent with growing evidence that digital game interventions can improve knowledge, attitudes, and self-efficacy among young people. It should be noted that the current findings primarily reflect acceptability and perceived usefulness among youth and nonpregnant users; generalizability to pregnant adults—the intended target population—remains to be established. Future deployment strategies should consider differentiated approaches for these 2 populations: school-based or community programs for adolescent reproductive health literacy, and prenatal clinic integration for pregnant women seeking to navigate the pregnancy journey.

### Comparison With Prior Work

#### Serious Games and Gamification in Prenatal Care

Our findings align with a recent systematic review of randomized trials in prenatal health care showing that gamification-based educational interventions can improve maternal knowledge, attitudes, preventive behaviors, and anxiety management [4]. The review also highlighted methodological variability and implementation challenges, underscoring the need for simple, scalable, and theoretically grounded educational tools—an area in which our low-cost, narrative-based game may offer practical advantages. Specific randomized trials, such as Tobbstop, which improved smoking cessation rates among pregnant women [7], provide further evidence that serious games can positively influence pregnancy-related health behaviors. These studies support the potential of game-based learning in facilitating decision-making and risk-reducing behaviors during pregnancy, consistent with our findings regarding knowledge acquisition and user engagement.

#### Serious Games for Women’s Health

Scoping reviews of serious virtual games for women’s health education have emphasized persistent gaps, including limited integration of educational theory, inadequate user involvement, and a scarcity of games that address psychosocial and administrative dimensions across the continuum of women’s health [11]. A systematic review of serious gaming in women’s health care highlighted its potential for clinical skill development among health care professionals [16]. Our 9-chapter pregnancy narrative extends this line of work to a patient-facing context by presenting a longitudinal and holistic simulation of the pregnancy journey that incorporates both clinical and psychosocial factors. The structure of our game is informed by experiential learning frameworks and supports the integration of theory-driven educational design, which has been noted as lacking in prior research [17]. Educational game interventions for women have demonstrated benefits beyond obstetrics; for example, an online breast cancer awareness game significantly improved knowledge among female university students [18]. These findings suggest that narrative-based digital education may be broadly applicable to women’s health literacy, including pregnancy-related education.

#### Development Model and Cost-Efficiency

Our study demonstrates the feasibility of producing a functional, bilingual, visually rich, and widely accepted educational game using a low-cost clinician-student collaborative model. This approach differs from the design complexity and high resource requirements that are often reported for prenatal gamification interventions [4]. The collaborative model mirrors the participatory development strategies recommended in prior adolescent-focused digital education studies and aligns with broader trends in serious game design, which emphasize interdisciplinary teamwork, learner engagement, and iterative refinement [19]. This project suggests that universities, local governments, and health care teams can feasibly develop domain-specific educational tools without substantial financial investments or professional programming expertise.

#### Implications for Health Professions Education

Although only a minority of the participants were health care students or professionals, our findings are consistent with evidence from health care professional education. Prior studies have shown that serious games can enhance knowledge, skills, and attitudes while providing safe experiential learning environments [19]. Given the alignment of our game design with experiential learning principles and the positive reception of our narrative approach, adaptation to medical education, such as undergraduate obstetrics, midwifery training, or community health curricula, may be considered in relevant educational settings.

### User Feedback and Areas for Improvement

Although overall evaluations were positive, feedback from some users indicated a desire for increased gameplay interactivity or additional minigame components, reflected in the near-neutral interactivity score (mean 3.31, SD 1.10 and median 3, IQR 3-4). Similar critiques have been noted in women’s health serious game literature, where insufficient interactivity may limit engagement and educational impact [11]. Consistent with recommendations from prenatal gamification trials, future iterations may consider incorporating immersive minigames, nonlinear or branching decision paths, audio narration or animation, and adaptive learning sequences tailored to user characteristics. Enhancing gameplay depth may further strengthen educational engagement and potential behavioral impact, as suggested by previous serious game interventions for pregnancy and adolescent sexual health [20-22].

### Limitations

This study has several limitations. Survey participation was voluntary, raising the possibility of self-selection bias. The sample size (n=65) was modest and skewed toward teenagers. No preintervention or postintervention comparisons or control group designs were used. Platform analytics, particularly for Android, contained inconsistencies requiring reliance on the most stable snapshot. Long-term retention and behavioral outcomes were not evaluated, echoing the gaps identified in prior game reviews, including limited long-term follow-up and heterogeneous evaluation designs. Additionally, playtime data were self-reported via the survey rather than platform tracked; participants who abandoned the survey midway would not have contributed playtime data, precluding a meaningful correlation analysis between gameplay scores and missing playtime values.

### Future Directions

Future work may incorporate additional interactivity, minigames, and audiovisual enhancements to further enrich the learning experience. More rigorous evaluation designs, including pretesting and posttesting or randomized comparisons, are important to better quantify the educational impact. Localization and cultural adaptation may broaden international reach. Building on these findings, collaboration with schools and community health organizations could facilitate integration into adolescent reproductive health and perinatal education programs, in line with prior evidence supporting the feasibility and impact of serious games and digital interventions for youth health promotion.

### Conclusions

This study demonstrates that a low-cost, clinician-student collaborative serious game can deliver meaningful pregnancy education and achieve broad user engagement, including at the international level. The positive reception across platforms suggests that narrative-based simulations represent a promising approach to maternal health education and public outreach. Future research should incorporate preintervention and postintervention assessments or randomized comparisons and explore opportunities for localization, cultural adaptation, and integration into school-based or community reproductive health programs to enhance educational impact and broaden international applicability.

## Supplementary material

10.2196/93571 Multimedia Appendix 1 Subgroup analysis of gameplay evaluations and educational usefulness by age.

## Abbreviations

MANTRA: Maternal and Neonatal Training and Resilience Application
TIPS: Teaching Information and Prompt System
