# Supplementary material for: Development and Formative Evaluation of a Narrative-Based Serious Game for Pregnancy Education: Mixed Methods Study
Source: JMIR Form Res. 2026 Jul 13;10:e93571. doi: 10.2196/93571 (PMC13362874; doi:10.2196/93571)
Supplement: Multimedia Appendix 1 [file formative-v10-e93571-s001.docx]

| Domain | Item | Teenagers (n = 38)  Mean ± SE | Adults (n = 27) Mean ± SE |
| --- | --- | --- | --- |
| Gameplay evaluation | Game length | 3.10 ± 0.12 | 3.00 ± 0.12 |
|  | Game difficulty | 3.08 ± 0.12 | 3.00 ± 0.10 |
|  | Gameplay | 3.35 ± 0.11 | 3.37 ± 0.12 |
| Educational usefulness | Reduced anxiety | 4.08 ± 0.17 | 4.08 ± 0.18 |
|  | Educational usefulness | 4.30 ± 0.13 | 4.41 ± 0.13 |
|  | Knowledge acquisition | 4.30 ± 0.12 | 4.59 ± 0.10 |
|  | Story empathy | 4.35 ± 0.13 | 4.56 ± 0.10 |
|  | Overall satisfaction | 4.24 ± 0.16 | 4.48 ± 0.12 |

Supplementary Table S1. Subgroup analysis of gameplay evaluations and educational usefulness by age

Values are presented as mean ± standard error (SE).
